# The COVID-19 Pandemic: A Comprehensive Review of Taxonomy, Genetics, Epidemiology, Diagnosis, Treatment, and Control

**DOI:** 10.3390/jcm9041225

**Published:** 2020-04-24

**Authors:** Yosra A. Helmy, Mohamed Fawzy, Ahmed Elaswad, Ahmed Sobieh, Scott P. Kenney, Awad A. Shehata

**Affiliations:** 1 Department of Veterinary Preventive Medicine, Ohio Agricultural Research and Development Center, The Ohio State University, Wooster, OH 44691, USA; kenney.157@osu.edu; 2 Department of Animal Hygiene, Zoonoses and Animal Ethology, Faculty of Veterinary Medicine, Suez Canal University, Ismailia 41522, Egypt; 3 Department of Virology, Faculty of Veterinary Medicine, Suez Canal University, Ismailia 41522, Egypt; 4 Department of Animal Wealth Development, Faculty of Veterinary Medicine, Suez Canal University, Ismailia 41522, Egypt; ahmed_elaswad@vet.suez.edu.eg; 5 Department of Radiology, University of Massachusetts Medical School, Worcester, MA 01655, USA; ahmadsobeih@gmail.com; 6 Avian and Rabbit Diseases Department, Faculty of Veterinary Medicine, Sadat City University, Sadat 32897, Egypt; dr_awadali_1@yahoo.com; 7 Research and Development Section, PerNaturam GmbH, 56290 Gödenroth, Germany

**Keywords:** SARS-CoV-2, COVID-19, One Health, genetics, epidemiology, control, outbreak, pneumonia, treatment, diagnosis, public health

## Abstract

A pneumonia outbreak with unknown etiology was reported in Wuhan, Hubei province, China, in December 2019, associated with the Huanan Seafood Wholesale Market. The causative agent of the outbreak was identified by the WHO as the severe acute respiratory syndrome coronavirus-2 (SARS-CoV-2), producing the disease named coronavirus disease-2019 (COVID-19). The virus is closely related (96.3%) to bat coronavirus RaTG13, based on phylogenetic analysis. Human-to-human transmission has been confirmed even from asymptomatic carriers. The virus has spread to at least 200 countries, and more than 1,700,000 confirmed cases and 111,600 deaths have been recorded, with massive global increases in the number of cases daily. Therefore, the WHO has declared COVID-19 a pandemic. The disease is characterized by fever, dry cough, and chest pain with pneumonia in severe cases. In the beginning, the world public health authorities tried to eradicate the disease in China through quarantine but are now transitioning to prevention strategies worldwide to delay its spread. To date, there are no available vaccines or specific therapeutic drugs to treat the virus. There are many knowledge gaps about the newly emerged SARS-CoV-2, leading to misinformation. Therefore, in this review, we provide recent information about the COVID-19 pandemic. This review also provides insights for the control of pathogenic infections in humans such as SARS-CoV-2 infection and future spillovers.

## 1. Introduction

Coronaviruses are enveloped, single-strand RNA viruses that can infect a wide range of hosts including avian, wild, domestic mammalian species, and humans. Coronaviruses are well known for their ability to mutate rapidly, alter tissue tropism, cross the species barrier, and adapt to different epidemiological situations [1]. Six human coronaviruses have been reported since the 1960s; four of them (OC43, 229E, NL63, and HKU1) cause mild illness similar to the common cold and gastrointestinal tract infection. The other two, severe acute respiratory syndrome coronavirus (SARS-CoV) and Middle East respiratory syndrome coronavirus (MERS-CoV), have raised significant public health concerns due to their zoonotic emergence and crossing of the species barrier, causing high pathogenicity and mortality in humans [2]. SARS- and MERS-CoVs were reported to be transmitted from the main host (bats) to palm civets or dromedary camels, respectively, then finally to humans [3,4,5]. Both SARS- and MERS-CoVs were and are highly pathogenic, resulting in 8096 and 2519 human cases, with 9.6% and 34.3% fatality rate in 2003–2004 and 2012–present, respectively [6,7].

Several clusters of pneumonia cases of unknown causes were reported in Wuhan city, Hubei province, China, in December 2019. By epidemiological investigations, most of these patients were related to the Huanan Seafood Wholesale Market. The causative agent of this pneumonia was confirmed as the severe acute respiratory syndrome coronavirus-2 (SARS-CoV-2), previously named 2019 novel coronavirus (2019-nCoV), and the diseases was termed coronavirus disease-2019 (COVID-19) [8,9]. Based on phylogenetic analysis, SARS-CoV-2 forms a distinct lineage with Bat-SARS-like coronaviruses that belong to the order *Nidovirales*, family *Coronaviridae*, genus *Betacoronavirus*, and subgenus *Sarbecovirus* [9]. SARS-CoV-2 shares 96.3%, 89%, and 82% nucleotide similarity with bat CoV RaTG13, SARS-like CoV ZXC21, and SARS-CoV, respectively, which confirms its zoonotic origin [10,11].

At the beginning of the outbreak, scientists thought that the disease was initially only transmitted from animals to humans, then only between people who are symptomatic, until the first human-to-human transmission case from an asymptomatic carrier was documented in Germany [10,12,13]. This is also now evidenced by cases of community spread in which no direct links between current patients and suspected COVID-19 carriers can be made. As of 13 April 2020, the disease has caused a worldwide pandemic in more than 200 countries, with more than 1,700,000 confirmed human cases and 111,600 deaths. As a result of the rapid spread of the virus, authorities worldwide have begun limiting international and domestic travel and large gatherings at educational institutions, restaurants, bars, etc.

SARS-CoV-2 is reported to be transmitted between humans through direct contact, aerosol droplets, fecal–oral route, and intermediate fomites from both symptomatic and asymptomatic patients during the incubation period [9,14]. The disease is characterized by fever, dry cough, dyspnea, and diarrhea in 20–25% of patients who do not exhibit upper respiratory signs such as sneezing or sore throat [8,13]. In severe cases, the disease is characterized by pneumonia, metabolic acidosis, septic shock, and bleeding [14,15].

Several control measures are being instituted by nations around the world to extinguish the SARS-CoV-2 pandemic, including the issuance of travel advisories or even flight bans to and from infected countries, strict quarantine measures and traveler screenings, implementation of mitigation measures by healthcare specialists, application of social distancing measures for schools and popular gatherings, strict personal hygiene such as frequent handwashing, and wearing face masks [16]. Currently, world public health authorities such as the Centers for Disease Control and Prevention (CDC), World Health Organization (WHO), and other global partners are trying to control and prevent the spread of SARS-CoV-2. Additionally, WHO issued a guide to managing the recent pandemic including instructions for the rapid detection of the disease, emergency treatment, application of prevention and control strategies, supportive therapy, and prevention of disease complications [15].

Due to misleading information that is circulating and knowledge gaps about the newly emerged SARS-CoV-2, this review provides the most recent information about SARS-CoV-2, with emphasis on (1) the current status of SARS-CoV-2 outbreaks, (2) evidence regarding its origin by phylogenetic analysis, (3) epidemiological characteristics required for efficient control strategies, (4) diagnosis of the disease, (5) current control strategies and effective therapies, and (6) challenges to control future epidemics like those caused by coronaviruses. This review also provides insights for the control of pathogenic infections in humans with novel coronaviruses and spillovers in the future.

## 2. History of Coronavirus Outbreaks

Coronaviruses have repeatedly evolved during the past 1000 years [17]. The first recovery of coronaviruses involved the identification of illnesses in animals followed by the isolation of infectious bronchitis virus (IBV) from chickens in 1937 [18] and murine hepatitis viruses (MHV) from mice in 1949 [19]. Pigs were found to carry a transmissible gastroenteritis virus (TGEV) in the United States in 1946 [19]. Human coronaviruses were first characterized in the 1960s from respiratory tract infections [20]. The two first isolated viruses were B814 and 229E [21,22]. Since then, several other coronavirus strains have been isolated from humans using tissue culture (OC16 and OC43) [23,24]. The number of identified coronaviruses has continued to increase significantly to include viruses of several additional animal species such as calves, dogs, cats, bats, sparrows, rabbits, and turkeys [25]. 

In 2002–2003, SARS-CoV caused a disease outbreak with deaths in 29 countries, most cases being in China and Hong Kong. The total number of reported cases was 8096, of which 774 died, corresponding to a 9.6% fatality rate [6], before the disease died out in part due to strict quarantine protocols. Based on the genome sequence, SARS-CoV appeared to be very closely related to another virus from Himalayan palm civets, from which it may have emerged [3]. Later, civets were considered an intermediate host for SARS-CoV, with bats as the natural host [5]. 

Hu et al. [26] conducted a five-year surveillance study of SARS-related coronaviruses isolated from horseshoe bats in Yunnan province, China, where 11 SARS-like CoVs were identified. Genome comparisons revealed high genetic diversity among these viruses in several genes, including *S*, *ORF3*, and *ORF8*. Despite the differences in S protein sequences, all 11 SARS-like CoVs are still able to use the same human angiotensin-converting enzyme-2 (hACE2) receptor, demonstrating a close relationship with SARS-CoV. Therefore, SARS-CoV likely emerged through recombination of bat SARS-like CoVs before infecting civets, from which the recombinant virus spread to humans, causing the SARS epidemic [5,26].

Ten years later, MERS-CoV emerged in Middle Eastern countries where the virus was transmitted to humans from dromedary camels [27]. As of January 2020, MERS-CoV has resulted in 2519 laboratory-confirmed cases and 866 deaths (34.3% fatality rate), with more than 80% of the cases reported from Saudi Arabia [7]. The human and camel MERS-CoV strains share more than 99% identity with variations (substitutions) located in the *S*, *ORF3*, and *ORF4b* genes [28]. Phylogenetically, MERS-CoV is very close to bat coronaviruses HKU4 and HKU5 [29]. A comprehensive analysis of the evolutionary relationships indicated that MERS-CoV may have originated from bats as a result of recombination events within *ORF1ab* and *S* genes [30,31]. To gain access into the cell, MERS-CoV uses the human dipeptidyl peptidase 4 (DPP4) receptor [32]. This is also the case for MERS-related CoVs isolated from bats in China, whose spike proteins are able to bind to the same receptor as MERS-CoV, confirming the possibility of a bat origin for MERS-CoV [33]. 

In December 2019, SARS-CoV-2 emerged in Wuhan City, China, causing severe respiratory illness and mortality (see the epidemiology section). Early studies reported that it may have evolved from bats, as revealed by phylogenetic analysis [9] and its high identity (96.3%) with the bat coronavirus RaTG13.

## 3. Coronavirus Taxonomy 

Coronaviruses are enveloped, icosahedral symmetric particles, approximately 80–220 nm in diameter containing a non-segmented, single-strand, positive-sense RNA genome of about 26–32 kb in size [34]. Coronaviruses (CoVs) are one of the largest groups of viruses that belong to the order *Nidovirales*, suborder *Cornidovirineae*, and family *Coronaviridae*. *Coronaviridae* is classified into two subfamilies, namely, *Letovirinae* and *Orthocoronavirinae*. *Letovirinae* includes the *Alphaletovirus* genus, while *Orthocoronaviridae* is further classified on the basis of phylogenetic analysis and genome structure into four genera: *Alphacoronavirus* (αCoV), *Betacoronavirus* (βCoV), *Gammacoronavirus* (γCoV), and *Deltacoronavirus* (δCoV), which contain 17, 12, 2, and 7 unique species, respectively (ICTV 2018). The most recent classification of the *Coronaviridae* is shown in Table 1. Corona in Latin means crown, and this name was attributed to the virus due to the presence of spike projections from the virus envelope that give it the shape of a crown under the electron microscope; Nido means nest and refers to the ability of the viruses of this order to make a nested set of subgenomic mRNA [25,35].

Coronaviruses infect a wide range of wild and domestic animals; α- and βCoVs infect mammals, while γ- and δCoVs primarily infect birds (Table 1). A human coronavirus (HCoV) was first isolated in 1960 from hospitalized patients who suffered from common cold symptoms and was named B814 [36]. So far, the seven different HCoVs that infect humans are 229E, NL63, which belong to α CoVs, and HKU1, OC43, SARS, MERS, SARS-CoV-2, which belong to βCoVs. In 2002–2003, a pandemic caused by SARS-CoV (lineage B βCoV) originated in China [37]. In the Middle East, MERS-CoV (lineage C βCoV) emerged in 2012 [27]. In 2019, a newly emerged SARS-CoV-2, closely related to bat SARS-related CoVs, was clustered with lineage B βCoV. Chan et al. [13] demonstrated that SARS-CoV-2 represents a distinct lineage in the subgenus *Sarbecovirus* (previously, lineage 2b of βCoV) [38].

Additionally, other coronaviruses have caused pandemic diseases in domestic and wild mammals and birds, leading to high mortality rates and severe economic losses. These viruses include IBV in chickens [40], Beluga whale coronavirus SW1 (BWCoV-SW1) [41], bat coronaviruses CDPHE15 and HKU10 (ICTV 2018), porcine epidemic diarrhea virus (PEDV), TGEV, and sudden acute diarrhea syndrome (SADS-CoV) [42]. 

## 4. SARS-CoV-2 Genome Organization 

Since the emergence of SARS-CoV-2 in Wuhan City, China, in December 2019, many laboratories have been working on sequencing the genome of the causative agent. As of 14 April 2020, there are a total of 7655 complete genomes from 67 countries in the Global Initiative on Sharing All Influenza Data (GISAID) database [43] (Table 2). A reference genome is now available in the NCBI genome database (29,903 nucleotide, Reference Sequence: NC_045512.3) [44]. To date, there are a total of 875 sequences including one RefSeq sequence and 768 complete genomes at NCBI.

SARS-CoV-2 is a monopartite, single-stranded, and positive-sense RNA virus with a genome size of 29,903 nucleotides, making it the second-largest known RNA genome. The virus genome consists of two untranslated regions (UTRs) at the 5′ and 3′ ends and 11 open reading frames (ORFs) that encode 27 proteins (Table 3). 

**Figure 1:**
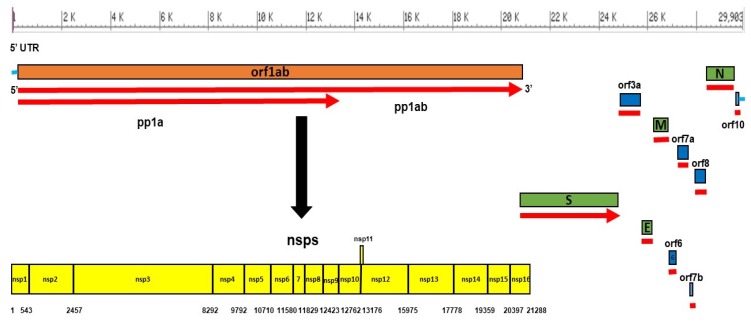
Genome organization of SARS CoV-2 and its encoded proteins. The *orf1ab* gene constitutes two-thirds of the genome, encodes a total of 16 non-structural proteins (NSPs) within the *pp1ab* gene, as shown in yellow, which are nsp1 (180 aa), nsp2 (638 aa), nsp3 (1945 aa), nsp4 (500 aa), nsp5 (306 aa), nsp6 (290 aa), nsp7 (83 aa), nsp8 (198 aa), nsp9 (113 aa), nsp10 (139 aa), nsp11 (13 aa), nsp12 (932 aa), nsp13 (601 aa), nsp14 (527 aa), nsp15 (346 aa), and nsp16 (298 aa). The other third of SARS CoV-2 includes four genes (in green) that encode four structural proteins (S, M, E, N), and six accessory genes (in blue) that encode six accessory proteins (orf3a, orf6, orf7a, orf7b, orf8, and orf10).

The first ORF (ORF1/ab) constitutes about two-thirds of the virus genome, encoding 16 non-structural proteins (NSPS), while the remaining third of the genome encodes 4 structural proteins and at least 6 accessory proteins. The structural proteins are spike glycoprotein (S), matrix protein (M), envelope protein (E), and nucleocapsid protein (N), while the accessory proteins are orf3a, orf6, orf7a, orf7b, orf8, and orf10, as shown in Figure 1 [2,13,38,45].

The 5′UTR and 3′UTR of SARS CoV-2 are comprised of 265 and 229 nucleotides, respectively. Orf1ab is 21,290 nucleotides and encodes either replicase proteins pp1a of 4405 amino acids (aa) (nsp1–nsp11) or pp1ab of 7096 aa (nsp1–nsp16), according to ribosomal frameshift. Of these proteins, (1) nsp1 suppresses the antiviral host response, (2) nsp3 is a papain-like protease, (3) nsp5 is a 3CLpro (3C-like protease domain), (4) nsp7 makes a complex with nsp8 to form a primase, (5) nsp9 is responsible for RNA/DNA binding activity, (6) nsp12 is an RNA-dependent RNA polymerase (RdRp), (7) nsp13 is confirmed as a helicase, (8) nsp14 is a 3′–5′ exonuclease (ExoN), 9) nsp15 is a poly(U)-specific endoribonuclease (XendoU). The remaining nsps are involved in transcription and replication of the viral genome [13,46,47]. 

## 5. Comparative Phylogenetic Analysis of SARS-CoV-2

Single-stranded RNA viruses exhibit a faster biological mutation rate due to the lack of proofreading activity of viral RNA polymerases [48]; however, unlike other mutation-prone RNA viruses, with the exception of the *Arenaviridae* family, CoVs do have limited proofreading capabilities, with the nsp14 protein allowing for the enhanced genome size of CoV family members [49].

Recombination is another mechanism of evolution in coronaviruses [50]. A high recombination frequency was demonstrated in murine hepatitis virus during mixed infection, where the majority of viruses recovered after three passages were recombinants [51]. Recombination was also reported for MERS-CoV and SARS-CoV. Seven putative recombination regions were detected in ORF1ab and S protein between SARS-CoV and six other coronaviruses by in silico analysis of their genomes [52]. Similarly, bioinformatic analysis of MERS-CoV genomic data revealed 28 recombinant sequences from humans and camels [53]. Recombination in SARS-CoV-2 is not yet clearly understood. Initial studies suggested that it may have occurred in the course of SARS-CoV-2 evolution [9], while other researchers excluded the possibility of recombination based on a full genome evolutionary analysis investigating putative recombination events [11]. 

**Figure 2:**
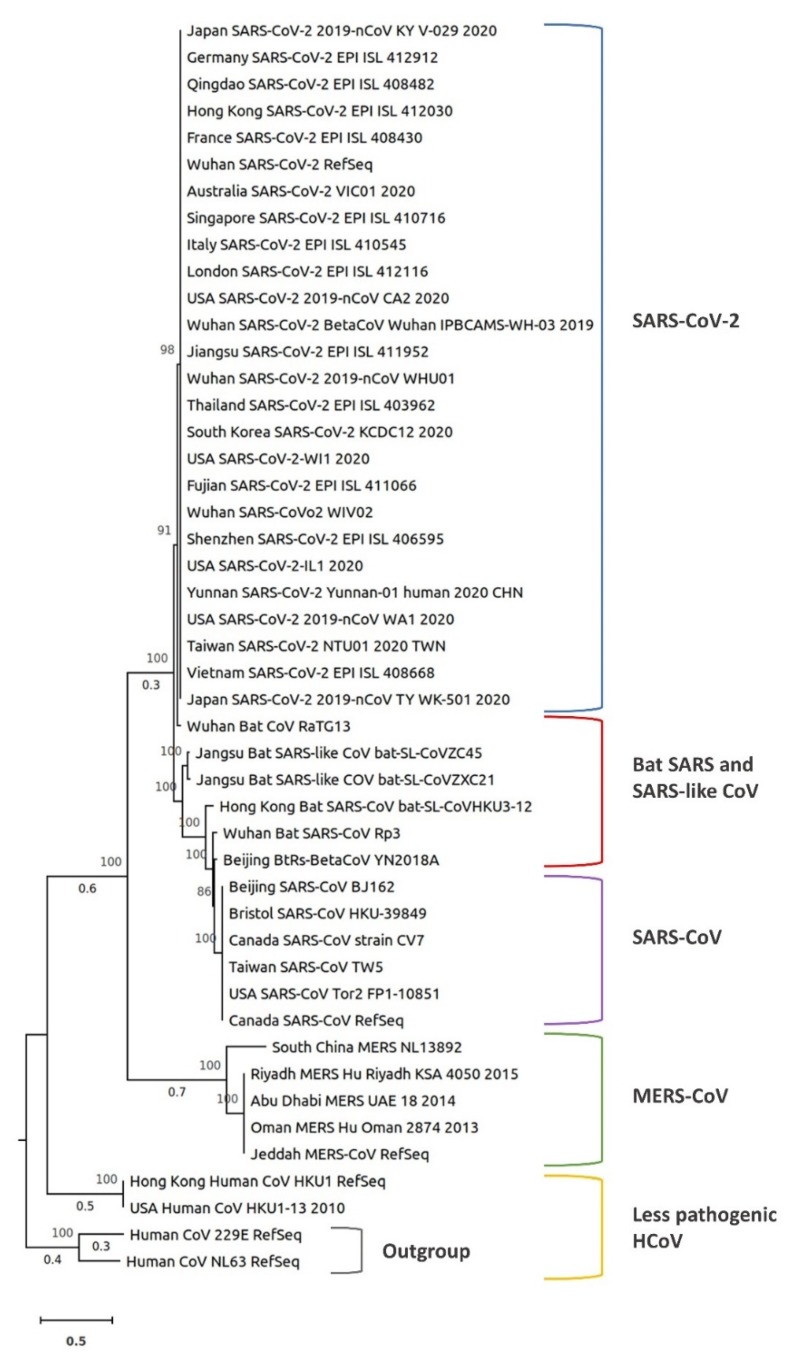
Phylogenetic tree based on the complete genome sequences of 45 selected coronaviruses from 18 countries including the SARS-CoV-2, SARS-CoV, HCoV, bat SARS, SARS-like CoV, and MERS-CoV. The tree was constructed in IQ-TREE using the maximum likelihood method, ModelFinder, and ultrafast bootstrap approximation (1000 replicates). The tree is drawn to scale, with branch lengths (numbers below the branches) measured in the number of substitutions per site. Branch lengths less than 0.3 are not shown. Numbers above the branches represent the percentage of replicate trees in which the associated viruses clustered together in the bootstrap test. The tree is rooted with two human coronavirus species from the genus *Alphacoronavirus* as an outgroup (HCoV-229E and HCoV-NL63).

To better understand the evolution of SARS-CoV-2, we performed a phylogenetic analysis of 45 representative coronaviruses from 18 countries including SARS-CoV, SARS-CoV-2, HCoV, bat SARS CoV, bat SARS-like CoV, and MERS-CoV. The viral genomes were obtained from the GISAID and NCBI databases. Multiple sequence alignment was performed using kalign 3 [54]. A phylogenetic tree was constructed based on whole-genome sequences (coding sequences of all genes) in IQ-TREE, using the maximum likelihood method, ultrafast bootstrap approximation, and ModelFinder [55,56]. The tree was drawn to scale, with branch lengths measured in the number of substitutions per site. The bootstrap values were determined by 1,000 replicates. The tree was visualized in MEGA X [57] (Figure 2). 

In the analysis we performed, all SARS-CoV-2 samples from the 18 countries clustered together and were close to bat SARS or SARS-like coronaviruses, with Wuhan bat CoV RaTG13 being the closest virus. In addition, MERS-CoV and human CoV HKU1 were very distant from SARS-CoV-2 (Figure 2). Within MERS-CoV samples, the South China MERS-NL13892 clustered separately from other MERS-CoVs. The two bat SARS-like CoVs (bat-SL-CoVZC45 and bat-SL-CoVZXC21) were the second closest viruses from bats to SARS-CoV-2 (Figure 2). All SARS-CoVs from China, Canada, England, and the US were in a single cluster.

Zhou et al. [9] conducted a phylogenetic analysis of SARS-CoV-2 against previously identified coronaviruses based on their whole-genome sequences, main structural protein genes, and non-structural protein genes. SARS-CoV-2 clustering was different depending on whether the whole genome or specific genes were used in the analysis. For example, SARS-CoV-2 clustered with the members of the subgenus *Sarbecovirus* including the SARS-CoV (79.5% identical) that caused the global pandemic in 2003 and other bat SARS-like viruses (96% identical at the whole-genome level), but the topological position within the Sarbecoviruses changed when individual genes (*ORF1ab*, *S*, *E*, *M*, and *N*) were used for clustering [2,9]. 

## 6. SARS-CoV-2 Mutations and Their Effects

Based on the whole-genome sequence alignment, SARS-CoV-2 shares 89% identity with bat SARS-like CoVZXC21, 82% with SARS-CoV, and 96.3% with bat CoV RaTG13 [11,13]. Alignment of the predicted protein sequences of SARS-CoV-2 to those of SARS-CoV or SARS-like coronaviruses revealed a total of 380 amino acid substitutions between these viruses [2]. These amino acid substitutions were distributed as follows: 348 mutations in nonstructural proteins (ORF1ab, 3a, 3b, 7a, 7b, 9b, and ORF14), 27 in S protein, and 5 in N protein. No amino acid substitutions were detected in E or M proteins, indicating that E and M proteins are highly conserved among these viruses. 

It has been reported that SARS-CoV-2 uses the same cellular receptor, hACE2, as SARS-CoV to gain entry into the cell [9,58,59]. The analysis of the receptor-binding domains (RBD) of SARS-CoV and SARS-CoV-2 S protein revealed similar binding affinities [60]. Wu et al. [2] found a total of 27 amino acid substitutions in the S protein but not in the receptor-binding motif (RBM) that directly interacts with hACE2, which may affect host tropism. These 27 substituted residues were distributed as follows: 17 in the S1 subunit [6 in the RBD and 6 in the subdomain (SD)] and 10 in the S2 subunit. Wan et al. [58] reported similarity in the spike protein RBD, including RBM, of both SARS-CoV and SARS-CoV-2, in addition to the presence of several residues in SARS-CoV-2 RBM that favor the interaction with human ACE2. These results agree with the genomic analysis of SARS-CoV-2, according to which the S2 subunit of the spike protein shares 99% identity with those of two bat SARS-like CoVs (SL-CoVZXC21 and ZC45) and of human SARS-CoV [13]. While the SARS-CoV-2 S2 subunit was conserved, the S1 subunit shares an overall 70% identity with those of bat and human SARS-CoV. The RBD core domain of S1 is highly conserved, with most of the amino acid differences located in the external subdomain that is responsible for the direct interaction with host receptors [13]. Investigators have also reported the presence of a polybasic cleavage site and predicted O-linked glycans that are unique to SARS-CoV-2 S protein. Differences in SARS-CoV-2 S protein and the high contagious nature of this virus suggest that SARS-CoV-2 has evolved via natural selection for binding to human ACE2 receptor [61].

ORF3b also differs in SARS-CoV-2. ORF3b deletion mutations in SARS-CoV do not affect viral replication in vitro [62]. ORF3b may play a role in viral pathogenicity in addition to its inhibitory effects on interferon (IFN) expression and signaling [63,64]. Recently, a novel short putative protein was identified in ORF3b of SARS-CoV-2 [13]; however, the function of this novel protein is still not known. SARS-CoV-2 ORF8 is closer to those of bat SARS CoV ZXC21 and ZC45 and distant from that of human SARS-CoV [13].

## 7. Genetic Diversity of SARS-CoV-2

The assessment of genetic diversity among 86 complete or semi-complete genomes of SARS-CoV-2 viruses revealed three deletions in the genome of isolates from Japan, USA, and Australia in addition to many other substitution mutations. The deletion mutations were in the *ORF1ab* gene (3-nucleotide and 24-nucleotide deletion) and at the 3′ end of the genome (10-nucleotide deletion). Of the 93 substitution mutations, 42 changed the amino acid sequence of structural and non-structural proteins [65]. The 3- and 24-nucleotide deletions in *ORF1ab* are expected to reduce the protein sequence by 1 and 8 amino acid residues, respectively, without changing the reading frame, but the functional effects have yet to be investigated. 

The alignment of SARS-CoV-2 reference S protein gene against all SARS-CoV-2 sequenced genomes from China, USA, Japan, Australia, and Taiwan revealed 99.97–100% identity, with 100% query coverage (also confirmed by our phylogenetic analysis, Figure 2), while the identity and coverage for SARS-CoV S protein gene were 74.5% and 91%, respectively. Also, the S protein gene from bat SARS and SARS-like coronavirus isolates shared 76.5–83% identity with that of SARS-CoV-2. This agrees with previous conclusions regarding the evolutionary analysis of SARS-CoV-2 [11,44]. In the phylogenetic analysis we performed, SARS-CoV-2 viruses were in the same cluster regardless of the geographic region (Figure 2). These results strongly suggest the possibility of a recent common ancestor for all SARS-CoV-2 or the transmission of the same virus strain across countries. 

## 8. Epidemiology of COVID-19

The outbreak of COVID-19 originated from Wuhan City, Hubei province, in China. Fifty-five percent of the infected cases before 1 January 2020 were linked to the Huanan Seafood Wholesale Market. However, the first human-to-human case of SARS-CoV-2 infection reported on 1 December 2019 did not have any exposure to this market [66,67]. In mid-January 2020, SARS-CoV-2 spread to other provinces of China due to the Spring Festival travel season. SARS-CoV-2 was transmitted from China to other countries via international travelers. On 13 January 2020, the first case of SARS-CoV-2 infection was confirmed outside China in Thailand, and on 16 January 2020 the first infected case was confirmed in Japan. These cases were also linked to the Huanan Seafood Wholesale Market. By 25 January 2020, the number of confirmed cases had risen to 2062, including 2,016 in China, Thailand, Hong Kong, Macau, Australia, Malaysia, Singapore, France, Japan, South Korea, Taiwan, the US, Vietnam, Nepal, and Sweden. On 30 January 2020, China reported a sharp rise in the number of infected cases, with the presence of infection in more than 18 countries. Therefore, WHO declared the SARS-CoV-2 outbreak to be a Public Health Emergency of International Concern [68]. 

As of 16 March 2020, more than 150 countries and territories have been affected, with major outbreaks in central China, South Korea, Italy, Iran, France, and Germany [69]. There were 167,511 confirmed cases of SARS-CoV-2 infections, with 6606 deaths and about 8% estimated mortality rate. More than 73% of these cases have been reported in mainland China [69]. At this time, the number of global cases has shown a drastic increase within a short time, confirmed cases and deaths in China have not increased too much, while confirmed cases and deaths in other countries have drastically increased (Table 4). The number of confirmed cases increased from 2798 to 17,391 in one week (between 27 January and 3 February), and the number of infected countries doubled (from 12 to 24). Due to the rapid increase of the number of infected cases and infected countries, the WHO declared SARS-CoV-2 a pandemic on 11 March 2020 and on 13 March 2020, the WHO declared Europe to be the new center of the pandemic due to the massive increase of confirmed cases there [70]. On 23 March 2020, Italy reported the highest number of deaths (5560) followed by China (3276), Spain (1720), and Iran (1685). One week later (30 March 2020), the global map of COVID-19 had changed. For example, the highest number of cases was reported in the USA (122,653 cases; 2112 deaths) followed by Italy (97,689 cases; 10,781 deaths), China (82,447 cases; 3310 deaths), Spain (78,797 cases; 6528 deaths), Germany (57,298 cases; 455 deaths), France (39,642 cases; 2602 deaths), and Iran (38,309 cases; 2640 deaths). As of 6 April 2020, there were 1,210,956 confirmed cases of SARS-CoV-2 infection (most of the cases, 307,318, were in the USA) and 67,594 deaths (most of the deaths, 15,889, were in Italy). One week later (13 April 2020), the number of confirmed cases of SARS-CoV-2 increased 1.7 times (up to 524,514 confirmed cases), and the number of deaths increased 2.5 times (up to 20,444 deaths) in the USA alone. The number of confirmed cases, deaths, and infected countries are shown in Table 4.

### 8.1. Source of Infection and Evolution of SARS-CoV-2

The origins of more than 75% of coronavirus infections are considered zoonotic, i.e., animals are the main source of the outbreaks. For example, SARS-CoV was transmitted from palm civets to humans, and MERS-CoV from dromedary camels to humans. Bats are currently considered a reservoir for all human coronaviruses, as mentioned above [5,71]. Many coronaviruses are circulating in animals but have not yet infected humans. The type of animal that SARS-CoV-2 originated from is still unclear. At the beginning of the outbreak in Wuhan, China, many patients were linked to the Huanan Seafood Wholesale Market, suggesting animal-to-person spread. After retrospectively studying case reports, the number of patients that did not have exposure to animal markets has risen, indicating person-to-person spread was also occurring at that time [66]. SARS-CoV-2 is closely related to bat coronaviruses and SARS-CoV [8]. A group of researchers reported early in the outbreak that the novel SARS-CoV-2 has the highest similarity of codon usage bias with snakes [72,73]; however, this method to determine initial host origins is dubious. Interestingly, researchers also reported one amino acid difference in the receptor-binding domain of the S protein of Pangolin-CoV compared to that of SARS-CoV-2, suggesting that pangolins might play a role as an intermediate host (Xiao et al., data currently under review). Another group of researchers reported that the virus originated from bats based on the genome sequence of SARS-CoV-2, which is 96% identical to bat coronavirus RaTG13. There were speculations that SARS-CoV-2 is a laboratory-engineered CoV and leaked directly from a laboratory in Wuhan where a bat CoV (RaTG13) was recently reported. However, there is no evidence to support this allegation [74]. Recently, a group of researchers found that SARS-CoV-2 replicates poorly in dogs, pigs, chickens, and ducks but efficiently in ferrets and cats [75]. Scientists are still trying to find the main source of the disease outbreak and identify the definitive intermediate hosts.

### 8.2. Mode of SARS-CoV-2 Transmission to Humans: Transmission Dynamics and Virus Persistence

**Figure 3:**
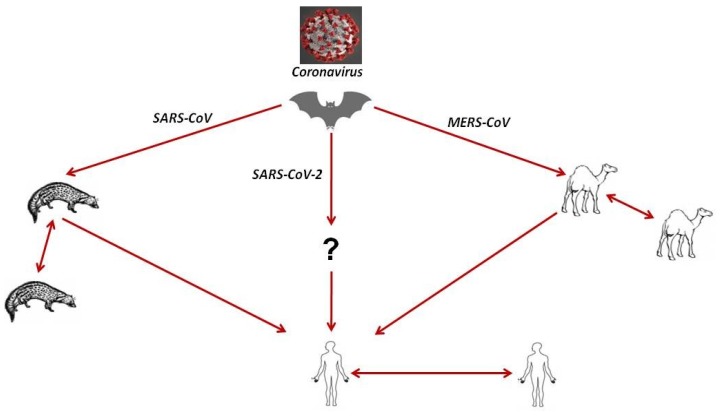
The transmission cycle of coronaviruses including MERS-CoV, SARS-CoV, and SARSCoV-2. The transmission of the virus to humans occurs by direct contact with infected animals. The continuous line represents direct transmission.

Both established (SARS-CoV, MERS-CoV) and novel (SARS-CoV-2) coronaviruses were reported to spread from an infected person to a non-infected person through direct or indirect contact. SARS-CoV-2 infection was reported to be transmitted directly from person to person like most respiratory viruses via close contact with an infected person or through respiratory droplets (aerosol) produced when an infected person coughs or sneezes. These droplets can be inhaled to reach the lung. The virus can be indirectly transmitted via touching a surface or an object that was previously contaminated with the virus and then touching the face, eyes, or mouth [76] and possibly via the fecal–oral route [77,78]. Asymptomatic carriers (during the incubation period of the virus) and patients after recovery from the acute form of the disease are also considered a potential source of virus transmission to healthy persons [10,12]. Interestingly, human coronaviruses are able to survive on steel, metal, wood, aluminum, paper, glass, plastic, ceramic, disposable gowns, and surgical gloves for 2–9 days. High temperature (≥30 °C) can reduce the persistence period, while low temperature (4 °C) increases the persistence time up to 28 days [79]. Transmission of the virus vertically from mother to fetus or via breast milk has not been confirmed yet [80]. The transmission cycle of coronavirus among animals and humans is shown in Figure 3.

### 8.3. Risk Factors for SARS-CoV-2 Infection and Their Assessment

There are many factors that affect SARS-CoV-2 transmission and spread. These factors include, but are not limited to: (1) travel to or contact with individuals who have recently visited Wuhan, China, or other places experiencing an outbreak; (2) close contact with persons who are diagnosed positive for the disease, such as healthcare workers caring for patients with SARS-CoV-2; (3) contact with droplets and secretions (produced by sneezing or coughing) from an infected person and eating or handling wild animals native to China such as bats. Additionally, the risk of infection is higher for the elderly and for patients suffering from pre-existing illnesses such as cardiovascular disease, hypertension, diabetes, and chronic respiratory disease [66]. The reported fatality rate based on age is 14.8% for people ˃80 years of age, 8% for people between 70 and 79 years, 3.6% for people between 60 and 69 years, 1.3% for people between 50 and 59 years, 0.4% for people between 40 and 49 years, 0.2% for people between 10 and 39 years; no fatalities have been reported for children under 10 years of age. Notably, the fatality rate is higher in males (2.8%) than in females (1.7%) [81,82]. 

### 8.4. Clinical Characteristics and Susceptibility of SARS-CoV-2 Infection in Humans

The estimated incubation period of the novel coronavirus ranges from 2 to 14 days. However, some cases had an incubation period of 21, 24, or 27 days [83]. The complete clinical picture of SARS-CoV-2 is still unclear. The disease begins with flu-like symptoms that include fever, fatigue, dry cough, sore throat, shortness of breath, headache, chest tightness, chest pain, and muscle pain. Some of SARS-CoV-2 patients have runny nose, nausea, vomiting, and diarrhea [84]. People can be infected without showing symptoms, which allows the virus to spread more effectively from person to person. Complications can occur due to COVID-19 leading to severe infections, such as pneumonia (infection of the lungs), kidney failure, and death [8]. The mild phase of the disease can last up to 2 weeks, while severe or critical disease lasts approximately 3 to 6 weeks (this analysis was conducted on 55,924 confirmed cases). Additionally, the time from the disease onset to the development of severe disease is one week, while the time from the onset of symptoms to death ranges from 2 to 8 weeks [82].

Based on the data analysis of 72,314 confirmed cases of SARS-CoV-2 in Wuhan City, China, by 11 February, 80.9% of the cases were mild with flu-like symptoms, and patients recovered at home, 13.8% were severe with pneumonia and shortness of breath, 4.7% were critical with respiratory failure and septic shock resulting in organs failure, and approximately 2% of the cases were fatal [85]. Another study was conducted on 99 hospitalized patients, and symptoms were classified as follow: fever (83%), cough (82%), shortness of breath (31%), muscle ache (11%), confusion (9%), headache (8%), sore throat (5%), runny nose (4%), chest pain (2%), diarrhea (2%), and nausea and vomiting (1%) [8]. 

## 9. Diagnosis of SARS-CoV-2 Infection 

The rapid diagnosis of SARS-CoV-2 infection is the cornerstone of disease control. It depends on several criteria including case history, clinical symptoms, serology, molecular diagnosis, and computed tomography (CT) imaging. On 2 March 2020, WHO published interim guidance for laboratory testing of suspected human cases, with precautions for specimen collection, packing, shipment, and amplification of nucleic acid to detect viral genes (*N*, *E*, *S*, and *RdRp*) [82]. SARS-CoV-2 uses the same cell entry receptor, hACE2, as SARS-CoV. Therefore, oral swabs, bronchoalveolar lavage fluid (BALF), blood, as well as anal swabs are the best samples used for virus diagnosis [86]. A proper diagnosis depends primarily on the factors described below.

### 9.1. Epidemiological History

The strict monitoring of case history in clinically suspicious patients is considered the first step in the early diagnosis of SARS-CoV-2 infection. Clinically suspicious patients are those who suffer from fever and lower respiratory tract infection symptoms (for details, see the clinical characteristics section) and reside within or have traveled to endemic regions or had close contact with a confirmed or suspected case. Additionally, SARS-CoV-2 can be transmitted by symptomatic and asymptomatic patients especially to the high-risk group mentioned above (for details, see the risk assessment section) [13].

### 9.2. Laboratory Diagnosis

The blood profiles of patients suffering from SARS-CoV-2 infection revealed the following: (1) increased C-reactive protein and erythrocytes, (2) increased myohemoglobin, liver enzymes, and muscle enzymes, with a high level of D-dimer in severe cases, and (3) normal or decreased white blood cell counts and lymphocytes in the early stage of the disease, with advanced lymphocytopenia in severe cases [13]. In ICU patients, high levels of plasma granulocyte colony-stimulating factor (GCSF), IP10, IL2, IL7, IL10, TNF-α, and MIP1a were reported [38]. 

### 9.3. Virus Detection

Electron microscope examination of SARS-CoV-2 revealed the typical coronavirus morphology. Further, SARS-CoV-2 was successfully isolated from human respiratory epithelial cells or BALF samples of infected patients using Huh7 cells and Vero E6 cells. The isolated strain was confirmed by immunofluorescent antibody techniques using the cross-reactive nucleoprotein (NP) antibody. Serum neutralization tests (SNT) using Vero E6 cells were conducted to confirm the neutralization activity in IgG-positive viral samples [87].

### 9.4. Serological and Molecular Diagnosis

IgM and IgG ELISA detection kits using bat SARSr-CoV Rp3 NP were developed with no cross-reaction against human coronaviruses except SARSr-CoV [80]. Using these serological tools, viral antibody titers were increased in SARS-CoV-2-infected patients [38]. The procedures of ELISA for the determination of SARS-CoV-2 IgG were described before [86]. Nucleic acid detection is the main, fastest, and most sensitive test for the diagnosis of SARS-CoV-2 infection. Recently, two nested RT-PCR and two real-time RT-PCR assays have been developed with successful detection of the first 25 positive cases of infection in Japan [88]. Three real-time RT-PCR techniques have been designed based on the *E*, *RdRp*, and *N* genes [89]. Also, scientists established molecular detection tools for SARS-CoV-2 based on the *S* gene [86]. 

### 9.5. Radiological Diagnosis

**Figure 4:**
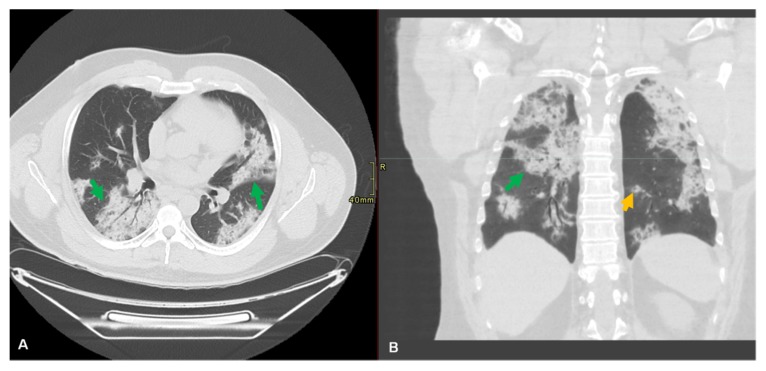
Lung of **a** 51-year-old male patient with a history of hepatitis C and symptoms of dry cough and shortness of breathing for three weeks. No recent travel or known contacts with infected subjects. Axial (**A**) and coronal computed tomography (CT) (**B**) of chest without contrast revealed bilateral peribronchial and subpleural consolidative opacities noted throughout both lungs (green arrow). There were scattered nodular consolidative opacities in a peribronchial distribution (orange arrow). The patient tested positive for SARS-CoV-2 RNA.

Chest X-ray examination in the early stage of the disease shows interstitial changes and multiple small plaque shadows. Chest CT scans play an important role in the diagnosis of acute respiratory disease syndrome (ARDS) and pneumonia as well as in the early detection of lung parenchymal abnormalities in patients at risk and provide an impression of secondary infection (Figure 4). 

Assessing these lungs parenchymal abnormalities conveys disease severity to clinicians. Using artificial intelligence models in the future may be useful in mass screening, to allow risk prioritization and help to minimize turnaround time [90]. Pan et al. [91] conducted a retrospective study to elaborate the time course of lung changes during recovery from infection. They described findings using international standard nomenclatures such as ground-glass opacity (GGO), consolidation, and crazy paving patterns. They established a semi-quantitative scoring system of 5 grades to quantify the degree of involvement based on an area ranging from 0% to >75%. The total score ranged from 0 to 25 (max), and involvement was subpleural, random, or diffuse. They found that in early stages (0–4 days after the onset of symptoms), GGO was the main finding in lower lung lobes; in progressive stages (5–8 days), the progression of lung disease involved three patterns of ground-glass, consolidation, and crazy paving, while in peak stages (9–13 days), dense consolidation became the prevalent feature; in absorption stages (>14 days), GGO was detected with no crazy paving and resolution of consolidations [91]. More than 75% of SARS-CoV-2-affected patients suffered from bilateral lung involvement, and 71% have multilobe involvement. CT examinations of 21 patients showed 29% consolidation and 86% GGO in the chest [8,9,92,93]. Another study examined 51 cases by CT and reported that 77% showed pure GGO, 75% exhibited GGO with reticular and/or interlobular septal thickness, 59% had GGO with consolidation, while 55% revealed pure consolidation. Bilateral lung involvement was reported in 86% of cases; in 80% of the cases the posterior part of the lung was involved, while in 86%, the periphery was involved [94].

## 10. Control and Treatment of COVID-19 Infection 

In January 2020, the WHO issued guidance for the clinical management of SARS when SARS-CoV-2 infection was suspected. In this guidance, the start of emergency treatments, immediate implementation of prevention and control strategies, early supportive therapy and prevention of SARS-CoV-2 complications were described in detail [15]. So far, there are no approved specific antiviral drugs for SARS-CoV-2 infection. Therefore, preventive measures and inactivation of the virus are essential to stop and control the spread of the disease. Human coronaviruses can be inactivated using 0.5% hydrogen peroxide, 62–71% ethanol, 0.1% sodium hypochlorite, 0.7–1% formaldehyde, 2% glutaraldehyde, or 0.23% povidone iodine within 1 minute. Other disinfectants such as 0.02% chlorhexidine digluconate, 0.55% orthophtalaldehyde, or 0.05–0.2% benzalkonium chloride are less effective [79]. 

In light of the urgent clinical demand, many drugs are approved to be used for clinical trials against SARS-CoV-2 infection, such as lopinavir/ritonavir, arbidol, interferon-alpha, favipiravir, chloroquine phosphate, darunavir/cobicistat, oseltamivir, and methylprednisolone. The most used antiviral drugs [95] are summarized in Table 5.

Generally, coronaviruses are not sensitive to current antiviral drugs, and high concentrations of drugs effective on these viruses cannot be used in vivo. Therefore, combinations of different therapies have been used for the treatment of coronavirus infections [96]. Some drug combinations that could be successful for the treatment of SARS-CoV-2 patients are lopinavir and ritonavir [97,98], lopinavir/ritonavir plus arbidol [99], and ribavirin and interferon [100,101]. The use of anti-inflammatory drugs such as glucocorticoids, IL-6 antagonist, janus kinase inhibitors (JAK), and choloroquine/hydrocholoroquine in SARS-CoV-2 patients is a dilemma, especially in patients suffering from an impaired immune system. Balancing the risk–benefit ratio is a critical issue. Corticosteroids may delay the elimination of the virus and increase the risk of secondary infection. In addition, drugs targeting pro-inflammatory cytokines can only inhibit specific inflammatory factors and thus may not be very effective in curbing the cytokine storm (excessive and uncontrolled release of pro-inflammatory cytokines). Moreover, some anti-inflammatory drugs such as JAK block INF-α production, which is important in fighting the virus [102]. 

Additionally, fecal transplantation was approved for clinical trials as a therapeutic option for SARS-CoV-2-related pneumonia based on the promising results obtained from fecal microbiota transplantation in patients suffering from antibiotic-associated diarrhea, active ulcerative colitis, and other viral infections [103,104,105,106]. Recently, it was found that intestinal microbiota-derived IFN in lung stroma confers protection against viral diseases such as avian influenza and respiratory syncytial virus [103]. Moreover, based on historical records of the effect of antiviral herbs on SARS and influenza H1N1, Chinese herbal formulas could be an alternative approach for the prevention of SARS-CoV-2 in a high-risk population [107], if no scientifically based therapeutics are available. It was found that *Sambucus formasana* Nakai exhibited a strong antiviral effect against human coronavirus NL63 [108].

## 11. Vaccination

To date, there is no vaccine to prevent SARS-CoV-2 infection, and trials for vaccine development are in the preliminary stages of research. Several vaccine candidates such as live attenuated, adenovirus-vectored, recombinant protein, and nucleic acid (DNA and mRNA) vaccines are in the pipeline [123].

## 12. Preventive Measures to Control the SARS-CoV-2 Spread 

The epidemiology of SARS-CoV-2 is still unclear, and data availability is limited. Therefore, it is imperative to follow preventive measures and safety precautions issued by health authorities to limit exposure to the virus and to reduce further spread. General hygienic measures should be implemented, such as (1) washing hands often with soap and water or an alcohol-based hand sanitizer, (2) cough or sneeze etiquette, recommending covering of the mouth, (3) avoiding touching eyes, nose, and mouth if the hands are not clean, (4) avoiding close contact with sick persons, (5) avoiding sharing dishes, glasses, bedding, and other household items with sick people, (6) cleaning and disinfection of surfaces that are often touched, and (7) staying home from work, school, and public areas when feeling sick. 

The transmission route of SARS-CoV-2 is probably not only through cough, respiratory droplets, and/or contaminated surfaces [13,124], but also through fecal–oral transmission [78]. Therefore, strict hygienic measures should be followed, especially in dense cities or agricultural spaces [125]. 

Since the SARS-CoV-2 spread is primarily driven by travel, screening of travelers who arrive at airports from pandemic areas for possible SARS-CoV-2 infection and entry-screening procedures are necessary. Also, general hygienic precautions during travel are highly recommended. Travelers who suffered from acute respiratory infection should be tested and reported to the respective public health authorities [73]. In addition, people should be motivated to notify and report about travel history and close contacts in case of SARS-CoV-2 infection.

Asymptomatic carriers (during the incubation period) and patients after recovery from the acute form are also considered potential sources of the virus [12,13]. Strict hygienic measures should be implemented to avoid virus transmission to healthcare workers and other contacts, i.e., placement of SARS-CoV-2 suspected or confirmed patients in single-person rooms and wearing personal protection equipment (PPE) such as masks, goggles, and protective gowns. Because early diagnosis and detection of asymptomatic carriers of SARS-CoV-2 are successful factors for the treatment and prevention of transmission, health authorities should designate laboratories to implement tests for a rapid and accurate diagnosis [126]. The control of coronaviruses is based on biosecurity regarding animals as well as on shifts in food habits, including discouraging the consumption of bushmeat and of animal products without appropriate cooking [127]. Ban of wet marketplaces where live or dead animals are handled should be implemented. Surveillance among people who have contact with wildlife and improvement of biosecurity regarding wildlife trade are urgently needed to prevent the next pandemic outbreak [128]. 

## 13. Challenges to Control SARS-CoV-2 and the Role of the One-Health Approach in Disease Control

The epidemiology of SARS-CoV-2 is still unclear. Many unresolved questions related to SARS-CoV-2 epidemiology and pathogenicity pose great challenges for researchers. These unresolved questions include: What is the origin of SARS-CoV-2? What is the intermediate host that transmitted the virus from bats? Why does the virus cause severe disease and mortality in the elderly or those with co-morbidities, while it is milder in children? Are aerosol, saliva, feces, urine, and foodborne the only routes of transmission? What are the other unknown routes of transmission? 

Control of the SARS-CoV-2 outbreak and future epidemics requires global efforts among medical and veterinary clinicians, diagnosticians, epidemiologists, public health experts, vaccinologists, pharmaceutical industries, economists, and governments to implement a One-Health approach [128,129]. These measures must include: (1) writing policies and supporting funds required for the implementation of One Health, prevention, and control measures, (2) hiring well-trained and professional personnel, (3) performing rapid and accurate diagnosis and treatment of infected persons, (4) developing and providing vaccines for virus control in humans, (5) conducting surveillance among wildlife for the identification and characterization of possible reservoirs and surveillance among people who are in contact with wildlife to identify risk factors in human behaviors and living environment, (6) improving hygienic measures, (7) assessing the social and economic impacts of COVID-19 on the population, (8) utilizing veterinary experience in the disinfection of premises and gatherings under the supervision of health authorities to decrease outbreaks in humans, (9) providing antiviral drugs for the treatment of the disease in humans, and (10) increasing public health awareness about the virus and its transmission.

## 14. Conclusions and Future Perspective

The SARS-CoV-2 outbreak started in Wuhan City, China, in December 2019. It is now a global pandemic, with 1,773,084 confirmed cases, 111,652 deaths, and 467,074 recoveries (as of 13 April 2020). The virus has the potential for rapid and extensive spread between people and countries. There are a lot of misleading information and knowledge gaps on the newly emerged SARS-CoV-2. Therefore, we reviewed the latest updates about different aspects including epidemiology, source of infection, transmission dynamics, zoonotic potential, virus characteristics, and discovery of novel strategies for disease control to avoid spillover of infection in the future. Bats play an important role in the transmission of the infection to humans. Coronaviruses are genetically diverse and have a high tendency towards frequent genetic mutations and gene recombination, which increases the risk of interspecies transmission. Information about the incubation period can help in establishing an effective quarantine for asymptomatic carriers, thus preventing the virus spread. From our perspectives and based on the currently available information about the virus and its epidemiology, the control of the SARS-CoV-2 requires an effective and global disease coordination effort including multidisciplinary research efforts (One-Health approach) through collaboration between governments, epidemiologists, virologists, health authorities, veterinarians, and physicians. At this stage of the disease outbreak, developing vaccines is crucial to limit the spread of the infection. 

## Figures and Tables

**Table 1:** Classification of *Coronaviridae* according to the International Committee of Taxonomy of Viruses (ICTV), with special emphasis on reservoir host, zoonotic importance, and major epidemics.

Family	Subfamily	Genus	Subgenus	Species	Abbreviations	Accession Numbers	Reservoir Host	Zoonotic [39]
***Coronaviridae***	Letovirinae	***Alphaletovirus***	*Milecovirus*	*Microhyla letovirus 1*				No
	***Orthocoronavirinae***	***Alphacoronavirus***	*Colacovirus*	*Bat coronavirus CDPHE15*	BtCoV-CDPHE15	NC_022103.1	Bats	No
			*Decacovirus*	*Bat coronavirus HKU10*	BtCoV-HKU10	NC_018871.1	Bats	No
				*Rhinolophus ferrumeguinum alphacoronavirus HuB-2013*	BtRfCoV-HuB13	KJ473807.1	Bats	No
			*Duvinacovirus*	***Human coronavirus 229E***	**HCoV-229E**	NC_002645.1	Human	No
			*Luchacovirus*	*Lucheng Rn rat coronavirus*	LRNV	NC_032730.1	Rats	No
			*Minacovirus*	*Ferret coronavirus*	FRCoV	NC_030292.1	Ferrets	No
				*Mink coronavirus 1*	MCoV	NC_023760.1	Minks	No
			*Minunacovirus*	*Miniopetrus batcoronavirus 1*	BtMiCoV-1	EU420138.1	Bats	No
				*Miniopetrus batcoronavirus HKU8*	BtMiCoV-HKU8	EU420139.1	Bats	No
			*Mytacovirus*	*Myotis ricketti alphacoronavirus Sax-2011*	BtMy-Sax11	NC_028811.1	Bats	No
			*Nyctacovirus*	*Nyctalus velutinus alphacoronavirus SC-2013*	BtNy-Sc13	NC_028833.1	Bats	No
			*Pedacovirus*	*Porcine epidemic diarrhea virus *	PEDV	NC_003436.1	Pigs	No
				*Scotophilus bat coronavirus 512*	BtScCoV-512	NC_009657.1	Bats	
			*Rhinacovirus*	*Rhinolophus bat coronavirus HKU2*	BtRhCoV-HKU2 (SADS)	NC_009988.1	Bats and pigs	Yes
			*Setracovirus*	***Human coronavirus NL63***	**HCoV-NL63**	NC_005831.2	Human	No
				*NL63-related bat coronavirus strain BtKYNL63-9b*	BtKYNL63	NC_032107.1	Bats	No
			*Tegacovirus*	*Alphacoronavirus 1*	TGEV CCoV FeCoV	NC_038861.1 KP_849472.1 JQ_408980.1	Porcines, canines, felines	No No No
		***Betacoronavirus***	*Embecovirus*	***Betacoronavirus 1***	**HCoV-OC43** **BCoV** **ECoV**	NC_006213.1 NC_003045.1 EF_446615.1	Human Bovines Equines	No No No
				*China Rattus coronavirus HKU24*	RtCoV-HKU24	NC_026011.1	Rats	No
				***Human coronavirus HKU1***	**HCoV-HKU1**	NC_006577.2	Human	No
				*Murine coronavirus*	MHV	NC_001846.1	Mouse	No
				Rabbit coronavirus HKU14	RbCoV HKU14	JN_874559	Rabbits	No
			*Hibecovirus*	Bat Hp-betacoronavirus Zhejiang2013	BtHpCoV-ZJ13	NC_025217.1	Bats	No
			*Merbecovirus*	Hedgehog coronavirus 1	EriCoV-1	NC_039207.1	Hedgehog	No
				**Middle East respiratory syndrome-related coronavirus**	**MERSr-CoV**	NC_019843.3	Human, camels, and bats	Yes
				Pipistrellus bat coronavirus HKU5	BtPiCoV-HKU5	NC_009020.1	Bats	No
				Tylonycteris bat coronavirus HKU4	BtTyCoV-HKU4	NC_009019.1	Bats	No
			*Nobecovirus*	Roussetus bat coronavirus GCCDC1	BtEoCoV-GCCDC1	NC_030886.1	Bats	No
				Roussetus bat coronavirus HKU9	BtRoCoV-HKU9	MG762674.1	Bats	No
			*Sarbecovirus*	**Severe acute respiratory syndrome-related coronavirus**	**SARSr-CoV**	NC_004718.3	Human, palm civets, and bats	Yes
			*Unclassified Betacoronavirus*	**Pangolin coronavirus**	**Pangolin-CoV**	NA_606875.1	Pangolins	No
		***Gammacoronavirus***	*Cegacovirus*	Beluga whale coronavirus SW1	BWCoV-SW1	NC_010646.1	Whale	No
			*Igacovirus*	Avian coronavirus	IBV	NC_001451.1	Birds	No
		***Deltacoronavirus***	*Andecovirus*	Wigeon coronavirus HKU20	WiCoV-HKU20	NC_016995.1	Birds	No
			*Buldecovirus*	Bulbul coronavirus HKU11	BuCoV-HKU11	NC_011547.1	Birds	No
				Coronavirus HKU15	PoCoV-HKU15	NC_039208.1	Pigs	No
				Munia coronavirus HKU13	MuCoV-HKU13	NC_011550.1	Birds	No
				White-eye coronavirus HKU16	WECoV-HKU13	NC_016991.1	Birds	No
			*Herdecovirus*	Night heron coronavirus HKU19	NHCoV-HKU19	NC_016994.1	Birds	No
			*Moordecovirus*	Common moorhen coronavirus HKU21	CMCoV-HKU21	NC_016996.1	Birds	No

Human coronaviruses (HCoVs) are in bold, while major epidemic-causing mammalian and avian viruses are in red.

**Table 2:** Number of completed genomes, partial sequences, or incomplete genomes of severe acute respiratory syndrome coronavirus-2 (SARS-CoV-2) from different countries submitted to the Global Initiative on Sharing All Influenza Data (GISAID) as of 14 April 2020.

Country	Number of Complete Genomes	Partial Sequences/Incomplete Genomes	Total
Algeria, Argentina, Czech Republic, Greece, Hungary, Saudi Arabia, Slovenia	3 *	0	3 *
Australia	391	0	391
Austria	21	0	21
Belarus, Columbia, Pakistan, Thailand, Turkey	2 *	0	2 *
Belgium	322	0	322
Brazil	36	0	36
Cambodia, Ecuador, Lithuania, Mexico, Nepal, Nigeria, Panama, Poland, South Africa, Sweden	1 *	0	1 *
Canada	129	0	129
Chile	7	0	7
China	346	47	393
Congo	42	0	42
Denmark, Mexico	9 *	0	9 *
Finland	40	0	40
France	204	0	204
Georgia	13	0	13
Germany	64	0	64
Ghana	15	0	15
Hong Kong	64	26	90
Iceland	601	0	601
India	32	1	33
Indonesia, Philippines	0	4 *	4 *
Iran	1	23	24
Ireland, South Korea	13 *	0	13 *
Italy	39	5	44
Japan	102	1	103
Kuwait, New Zealand, Vietnam	8 *	0	8 *
Latvia	5	0	5
Russia, Slovakia, Estonia	4 *	0	4 *
Luxembourg	86	0	86
Malaysia	7	3	10
Netherlands	585	0	585
Norway	29	0	29
Peru	1	1	2
Portugal	100	0	100
Senegal	23	0	23
Singapore	37	0	37
South Africa	6	0	6
Spain	105	0	105
Switzerland	52	0	52
Taiwan	22	0	22
United Kingdom	2540	1	2541
USA	1467	2	1469
Total	7655	118	7773

Available at: https://www.gisaid.org/, * Numbers are for each country.

**Table 3:** SARS CoV-2 genes and encoded polyproteins.

Gene	From	To	Gene Length (Nucleotide)	Protein	Protein Length (Amino Acid)
*5′ UTR*	1	265	265	Untranslated	–
*orf1ab*	266	21,555	21,290	pp1ab	7096
				pp1a	4405
*S*	21,563	25,384	3822	S	1273
*orf3a*	25,393	26,220	828	orf3a	275
*E*	26,245	26,472	228	E	75
*M*	26,523	27,191	669	M	222
*orf6*	27,202	27,387	186	orf6	61
*orf7a*	27,394	27,759	366	orf7a	121
*orf7b*	27,756	27,887	132	orf7b	43
*orf8*	27,894	28,259	366	orf8	121
*N*	28,274	29,533	1260	N	419
*orf10*	29,558	29,674	117	orf10	38
*3′UTR*	29,675	29,903	229	Untranslated	–

**Table 4:** Number of confirmed cases, deaths, and infected countries inside and outside China weekly [69].

Date	Number of Infected Countries and Territories	Cumulative Confirmed Cases			Cumulative Number of Deaths		
		Globally	China	Outside China	Globally	China	Outside China
13 December–6 January	1	44	44	0	0	0	0
13 January	2	45	44	1	1	1	0
20 January	4	282	279	3	6	6	0
27 January	12	2798	2741	37	80	80	0
3 February	24	17,391	17,238	153	362	361	1
10 February	25	40,554	40,235	319	910	909	1
17 February	26	71,429	70,635	794	1775	1772	3
24 February	30	79,331	77,262	2069	2618	2595	23
2 March	65	88,948	80,174	8774	3043	2915	128
9 March	105	109,577	80,904	28,673	3809	3123	686
16 March	152	167,511	81,077	86,434	6606	3218	3388
23 March	195	332,930	81,601	251,329	14,509	3267	11,242
30 March	204	693,282	82,447	610,835	33,106	3310	29,796
6 April	210	1,210,956	83,005	1,127,951	67,594	3340	64,254
13 April	213	1,773,084	83,597	1,689,487	111,652	3351	108,301

**Table 5:** Summary of drugs/treatments registered for clinical trials against SARS-CoV-2.

Drug/Treatment	Mode of Action	Antiviral Activity against COVID-19-Related Viruses	References
Lopinavir/Ritonavir	Protease inhibitor	- Provided good results against SARS-CoV decreased the viral load significantly and provided good results in COVID-19 patients	[97,98,109]
Arbidol	Inhibits membrane fusion	- Used for the treatment of influenza viruses in Russia and China - Lopinavir/ritonavir plus arbidol combination improved significantly the conditions of patients suffering from COVID-19 pneumonia	[102,110]
Interferon therapy	Inhibits many stages of virus replication: viral entry, transcription, replication, translation, assembly	- Opinavir/ritonavir plus interferon combination was used for the treatment of HIV infection - Ribavirin and interferon combination was used for the treatment of patients infected with MERS-CoV	[99,100,101]
Favipiravir	Inhibits viral RNA polymerase and mRNA capping	- Demonstrated an inhibitory effect on all influenza subtypes including neuraminidase- and M2 inhibitor-resistant strains - Showed inhibitory effects against Arenaviruses, Bunyaviruses, and Filoviruses	[111,112,113]
Chloroquine	Increases pH in host cell lysosomes and negatively influences virus–receptor binding, as well as interferes with the glycosylation of cellular receptors of SARS-CoV	- Exhibited a promising antiviral effect against SARS-CoV-2 in vitro - Improved COVID-19-pneumonia patients and shortened the course of the disease	[114]
Remdesivir	A monophosphoramidate of adenosine prodrug that incorporates into nascent viral RNA chains causing pre-mature termination	- Used against a wide range of RNA viruses such as Filoviridae, Paramyxoviridae, Pneumoviridae, and Coronaviridae; used successfully in COVID-19 treatment in the United States and showed no adverse events	[115]
Darunavir and Cobicistat	Inhibit 3 C-like protease (3CLpro).	- Used for the treatment of MERS-CoV in experimental animals - Used for the treatment of HIV-1 patients	[116]
Oseltamivir	Neuraminidase inhibitor	- Anti-influenza drug - Combination of nitazoxanide and oseltamivir is more effective in the treatment of ferrets infected with influenza virus compared to oseltamivir monotherapy	[97,117]
Steroid treatment (Methylprednisolon)	Anti-inflammatory	- Commonly used for the treatment of SARS patients suffering from severe pneumonia. - Because these drugs are immunosuppressive, they may delay viral clearance if given before viral replication is controlled	[102,118]
Convalescent plasma	SARS-CoV-2-neutralizing antibodies	- Immunotherapy combined with antiviral drugs is efficient against COVID-19	[119,120]
Mesenchymal Stem Cells	Anti-inflammatory and immunomodulatory	- Enhances recovery in COVID-19 patients.	[121]
Ivermectin	Anti-parasitic and antiviral	- Inhibits the in vitro replication of SARS-CoV-2 on Vero-hSLAM cells with 5000-fold reduction in viral RNA in 48 hours	[122]
